# Conducting an objective structured clinical examination under COVID-restricted conditions

**DOI:** 10.1186/s12909-024-05774-8

**Published:** 2024-07-25

**Authors:** Andrea Gotzmann, John Boulet, Yichi Zhang, Judy McCormick, Mathieu Wojcik, Ilona Bartman, Debra Pugh

**Affiliations:** Medical Council of Canada, 1021 Thomas Spratt Place, Ottawa, ON K1G 5L5 Canada

**Keywords:** Examination disruptions, OSCE, Performance assessment, Validity, Reliability

## Abstract

**Background:**

The administration of performance assessments during the coronavirus disease of 2019 (COVID-19) pandemic posed many challenges, especially for examinations employed as part of certification and licensure. The National Assessment Collaboration (NAC) Examination, an Objective Structured Clinical Examination (OSCE), was modified during the pandemic. The purpose of this study was to gather evidence to support the reliability and validity of the modified NAC Examination.

**Methods:**

The modified NAC Examination was delivered to 2,433 candidates in 2020 and 2021. Cronbach’s alpha, decision consistency, and accuracy values were calculated. Validity evidence includes comparisons of scores and sub-scores for demographic groups: gender (male vs. female), type of International Medical Graduate (IMG) (Canadians Studying Abroad (CSA) vs. non-CSA), postgraduate training (PGT) (no PGT vs. PGT), and language of examination (English vs. French). Criterion relationships were summarized using correlations within and between the NAC Examination and the Medical Council of Canada Qualifying Examination (MCCQE) Part I scores.

**Results:**

Reliability estimates were consistent with other OSCEs similar in length and previous NAC Examination administrations. Both total score and sub-score differences for gender were statistically significant. Total score differences by type of IMG and PGT were not statistically significant, but sub-score differences were statistically significant. Administration language was not statistically significant for either the total scores or sub-scores. Correlations were all statistically significant with some relationships being small or moderate (0.20 to 0.40) or large (> 0.40).

**Conclusions:**

The NAC Examination yields reliable total scores and pass/fail decisions. Expected differences in total scores and sub-scores for defined groups were consistent with previous literature, and internal relationships amongst NAC Examination sub-scores and their external relationships with the MCCQE Part I supported both discriminant and criterion-related validity arguments. Modifications to OSCEs to address health restrictions can be implemented without compromising the overall quality of the assessment. This study outlines some of the validity and reliability analyses for OSCEs that required modifications due to COVID.

## Background

Objective Structured Clinical Examinations (OSCEs) date back over five decades [1]. An OSCE is a standardized performance assessment, where standardized participants (SPs) interact with candidates on a series of scripted clinical scenarios, called cases or stations [2]. These performance-based examinations eventually became a mainstay of clinical skills assessment for certifying and licensing physicians [3]. In 1992, the Medical Council of Canada (MCC) introduced the Medical Council of Canada Qualifying Examination (MCCQE) Part II, a pre-requisite for medical licensure in Canada [4]. In 2004, passing the United States Medical Licensing Examination (USMLE) Step 2 Clinical Skills (CS) became a licensure requirement for all United States MD graduates and International Medical Graduates (IMGs) [3]. A similar performance assessment requirement for licensure was also introduced for osteopathic medical students in 2004 (COMLEX-USA-PE) [3]. Outside of North America, various other high-stakes performance-based assessments were also introduced [5–7]. These examinations were administered to ensure that graduating medical students, or those eventually seeking unrestricted medical licenses, possessed the skills needed to interact with and manage patients.

Performance-based examinations in medicine can take many forms but are generally constructed to measure data gathering (physical examination, history taking) and communication (with a patient or other healthcare provider). Other skills, including clinical decision-making, written communication, professionalism, and ethical behaviour have also been measured [8–10]. Scoring of the encounters can be done using checklists or rating scales (e.g., by the SP or an examiner in the room, or via videotape review), or some combination [11]. Although there can be considerable variation in the structure and content of OSCEs used for certification and licensure, researchers have provided ample evidence that the scores and pass/fail decisions are reliable and valid [12–14].

The psychometric properties of OSCEs and other performance-based assessments are well-described in the literature [15, 16]. Modeling clinical encounters based on typical reasons for visiting a physician and standardizing the exam administration helps ensure that the score interpretation is valid. If there are enough behavioral samples (i.e., SP interactions), and adequate rater training, reliable estimates of ability can be procured [17]. From an extrapolation perspective, several studies linked performance on OSCEs to future practice outcomes, including patient care and disciplinary actions [18]. Likewise, there are expected performance differences amongst defined candidate cohorts [19]. In support of the validity argument, candidates with more clinical experience and better language skills have higher average scores on some measured constructs [9]. For communication skills, including counselling and listening, women have outperformed men. As expected, scores from performance assessments measuring clinical skills were only weakly related to knowledge-based selected response examinations [20]. For decisions based on performance-based certification and licensure examinations, standards are typically established via defensible and properly implemented procedures [21]. When properly constructed and administered, OSCEs and other performance-based assessments used for credentialing and licensure of physicians can provide valid scores and associated pass/fail decisions [22].

### Interruptions in OSCEs during COVID

While national clinical skills assessments operated for over 30 years, the arrival of the coronavirus disease of 2019 (COVID-19) forced many testing organizations to rethink their administration protocols. The USMLE decided to cancel the Step 2CS examination and is attempting to measure some of the relevant clinical skills competencies in the other examinations required for medical licensure in the United States [23]. The National Board of Osteopathic Medical Examiners (NBOME) indefinitely suspended the COMLEX-USA-Level 2 Performance Evaluation (PE) and convened a Special Commission on Osteopathic Medical Licensure to investigate how clinical skills could best be measured [24]. The Special Commission proposed that, at least temporarily, enhanced attestation of clinical skills by the medical school would suffice. The MCC postponed the MCCQE Part II and attempted to pivot to a virtual format [25]. Unfortunately, the short time frame required to reestablish testing, the logistics of administering a virtual clinical skills assessment, and the large number of candidates to be tested, resulted in the MCC abandoning these efforts and cancelling the examination. Overall, the onset of COVID-19 had a drastic impact on performance testing. Given that previous research has indicated deviations to normal procedures can be a threat to score interpretation and the validity arguments for an examination [21, 22], it was necessary to validate new processes and procedures. The MCC was able to administer the National Assessment Collaboration (NAC) during this time frame, with modifications to content and delivery, to a large number of candidates.

### Background on the NAC examination

In 2011, the MCC introduced the NAC Examination, which is an OSCE [26] with the purpose of assessing the clinical skills of IMGs being selected to enter postgraduate training (PGT) programs across Canada. The NAC Examination is a requirement for IMGs to apply to a Canadian residency program and is used to assist Canadian medical school residency programs in their selection of candidates for PGT [26].

### NAC examination Pre-COVID

The NAC Examination consists of 10 different clinical scenarios per test administration and covers a variety of medical scenarios across various systems (e.g., endocrine, reproductive health) and disciplines (e.g., medicine, surgery). Each station has a combination of key feature checklist items, oral questions, and rating scales appropriate to the clinical scenario. Station scores are converted to percentages based on the aggregate item scores, and the total score is the average across the 10 stations. Total scores are adjusted across examination dates based on the difficulty of the set of stations [27] and converted to a reported score between 300 and 500. The NAC Examination was administered in early March 2020 and not disrupted as health restrictions came into effect later that month.

### Adjustments to the NAC examination during COVID

In September 2020 the MCC made modifications to the NAC Examination to ensure adherence to public health guidelines. These modifications were implemented for the administrations of the NAC Examination from September 2020 to September 2022[1]. To modify the NAC Examination, the MCC organized “work streams” which covered all areas of exam development and administration, such as registration, communications, personal protective equipment (PPE) and physical distancing, training, candidate orientations, delivery, content, and psychometrics. The major psychometric change was to reset the score scale and conduct a new standard-setting exercise for September 2020 to ensure that the pass/fail cut score was valid for this modified examination [28]. The new score scale was set to 1300 to 1500.

#### Delivery adjustments

Health Canada guidelines mandated no gatherings of large groups, so MCC adjusted all in-person training and orientation sessions to online modules. The MCC’s training and orientation stream created accessible, interactive online learning modules for all participants and, in addition, produced “cheat sheets” of quick reminders that could be delivered to the candidates and the physician examiners on exam day. Registration was done in waves so that not all participants arrived or left at the same time, and catering was delivered to individuals so that participants weren’t unmasking and eating in groups. Additionally, there was a reduction of shared touch points, including writing utensils, paper and personal belongings. All participants in the exam were required to use personal protective equipment (PPE), and sites were provided with masks, acrylic barriers, gloves and sanitizers. The exam spaces and materials were sanitized frequently, including doorknobs, chair arms and any laminated documentation the candidates were required to handle. Physical distancing was addressed by restricting the size of groups (administrators, SPs, candidates, physician examiners) and the use of visual reminders. The exam sessions also employed staggered start times to avoid large gatherings at breaks and at departure times.

#### Content adjustments

This “physically distanced” examination also required the adaptation of the exam content. These adjustments were guided by internal and external subject matter experts (SMEs). Through workshops with SMEs, the content team made iterative adjustments to the case content, scoring checklists and rating scales. They revised physical examination cases to include a “touchless” physical examination, where candidates verbalized their approach and their rationale, rather than demonstrating their skills on SPs. Physician examiners reported relevant findings to the candidate. As a necessary trade-off, the MCC assessed the candidates on their clinical reasoning skills related to physical examination, rather than their performance of the relevant maneuvers.

### Objectives of this study

This study aims to provide evidence to support the reliability and validity of the modified in-person NAC Examination with a touchless physical examination, as administered during the COVID-19 pandemic. The types of validity and reliability evidence that should be collected to support modified versions of an assessment are outlined, whether it is for a major modification or an interruption to exam delivery and content. With extensive modifications, the MCC was comfortable that the NAC Examination could be administered in-person and that relevant clinical skills could be assessed. The changes to the delivery and content required a new cut score to be established, as direct comparisons to pre- and post- COVID results cannot be supported. This study outlines outcome measures that should be evaluated when making major modifications to an exam program.

## Methods

The NAC Examination candidate study cohort, data sources (NAC Examination, MCCQE Part I), dependent variables (total scores and sub-scores), and independent variables (candidate demographics) are described in this section.

### Data sources

We analyzed data from the modified NAC Examination administered from September 2020 through October 2021[2]. Each candidate can challenge the exam up to 3 times, once per calendar year. If a candidate has a pass on the NAC Examination, they can challenge the exam again as their performance is used for residency selection purposes. The quantitative analyses described below were based on candidates who were attempting the NAC Examination for the first time (*n* = 2,433).

A new score scale and cut score are established when major delivery, content and scoring changes occur for an examination. Given the extensive content and delivery changes a new score scale and cut score was warranted. A new score scale was established in September 2020 along with a standard-setting exercise to establish a new cut score. Total scores for this score scale were equated across test forms to ensure that the comparison of scores and the cut score were on the same scale and therefore the candidate results during this time frame can be directly compared. Total scores ranged from 1300 to 1500 and sub-scores for Assessment and Diagnosis, Management, and Communication Skills were used for analyses.

The MCCQE Part I scores and subs-scores were used as criterion validity measures. The MCCQE Part I assesses the critical medical knowledge and clinical decision-making ability of a candidate at the level of a medical student completing their medical degree in Canada [29]. The MCCQE Part I is one of the requirements to obtain the Licentiate of the Medical Council of Canada, a credential that is required for medical license for various provinces and territories in Canada. It is a one-day computer-based exam consisting of 210 multiple-choice questions and a clinical decision-making component with 38 cases with short-menu and short-answer questions. The blueprint for the MCCQE Part I consists of two elements with sub-scores for *Dimensions of Care*: (1) Health promotion and Illness prevention, (2) Acute, (3) Chronic, and (4) Psychosocial aspects; and *Physician Activities*: (1) Assessment and diagnosis, (2) Management, (3) Communication, and (4) Professional behaviours. The blueprint was implemented in 2018; a new score scale, 100 to 400, was established at that time [30]. The MCCQE Part I is a co-requisite for IMG residency application, however, many candidates take the MCCQE Part I prior to attempting the NAC Examination as the MCCQE Part I is offered outside of Canada. We used this examination as criterion-related evidence for the NAC Examination as all candidates need to take both examinations for a residency application.

The data for the NAC Examination sample was merged with exam results for the MCCQE Part I taken in 2018 or later, resulting in 2,134 candidates having both exam scores. This matched sample was used to gather criterion-related validity evidence. The demographic variables include gender, type of IMG, previous PGT, and language. Threats to validity can occur when group differences are identified that cannot be explained. Gender, type of IMG, previous PGT and language are demographic variables that could support the valid score interpretation of NAC Examination total scores [9, 19]. As previous research indicates, gender, years of experience and language can lead to performance differences. We wanted to determine is this was also true for the NAC Examination. Gender was categorized as female or male based on information provided at registration. Type of IMG was categorized as Canadian Studying Abroad (CSA) and non-CSA; candidates were identified as CSAs if they indicated that they were Canadian citizens or permanent residents in Canada when entering medical school. All other candidates were categorized as non-CSA. The type of advanced education was categorized as PGT if the candidate had postgraduate training (all training was outside of Canada), and non-PGT if the candidate had no postgraduate training. Language was categorized as English or French based on the delivery language of the NAC Examination.

### Statistical analyses and outcome measures

#### Analyses

Several analyses were conducted to gather evidence to support the psychometric properties of the NAC Examination scores. Reliability evidence included Cronbach’s alpha, decision consistency, and decision accuracy ranges for the September 2020 to 2021 exams. Validity evidence included comparisons of differences for total scores and sub-scores for demographic groups: gender (male vs. female), type of IMG (CSA vs. non-CSA), PGT (no PGT vs. PGT), and the language for test administration (English vs. French). Criterion relationships were quantified through correlations of the total scores and sub-scores internal to the NAC Examination as well as with the MCCQE Part I.

##### Reliability analyses

Cronbach’s alpha was used to estimate score reliability for each test form. Cronbach’s alpha indicates the desired consistency (or reproducibility) of exam scores across replications of measurement [31]. Estimates indicating the decision consistency (DC) and decision accuracy (DA) of pass/fail decisions were calculated using the Livingston and Lewis procedure [32]. DC is an estimate of the agreement between classifications on potential parallel test forms; DA is the estimate of agreement between the observed classifications of candidates and those based on their true score (i.e., observed score ± measurement error). Ideally, both values should be high (i.e., 0.80 and above), suggesting reliable pass/fail classifications.

##### Validity analyses

Several separate t-tests were completed based on total score differences on the NAC Examination for the following demographic variables: (1) gender, (2) type of IMG, (3) PGT, and (4) language. Since several t-tests were conducted, we adjusted the significance level from 0.05 to 0.01 to guard against type I error [33]. For significant t-tests, Cohen’s *d* was also calculated [34]. Effect sizes of 0.20 are considered small, 0.50 medium, and 0.80 large.

Multivariate analyses of variance (MANOVA) were conducted for the same demographic analyses using the three NAC Examination sub-scores as the dependent variables: (1) Assessment and Diagnosis, (2) Management, and (3) Communication Skills. Analysis of variance (ANOVA) step-down tests were conducted for significant MANOVA results. We used a *p* < .01 for the MANOVAs to control for type I errors.

For criterion-related validity analyses, we calculated Pearson correlations between the NAC Examination total scores and sub-scores and their relationships with the MCCQE Part I total scores and sub-scores.

## Results

The descriptive statistics for the NAC Examination sample of 2,433 are shown in Tables 1 and 2. Table 1 shows the number of candidates, mean, and standard deviation (SD) for the total scores and the three sub-scores. Table 2 shows the number of candidates, mean, and SD for the total scores and sub-scores for each of the four demographic groupings of gender, type of IMG, type of PGT, and language.

**Table 1:** Descriptive statistics of the NAC Examination, total scores and sub-scores

	*N*	M	SD
Total Scores	2433	1397.9	25.7
Assessment and Diagnosis sub-score	2433	59.8	11.3
Management sub-score	2433	54.7	14.5
Communication Skills sub-score	2433	68.0	10.4

**Table 2:** Descriptive statistics for gender, type of IMG, PGT training, and language

Gender	Female			Male		
	*N*	M	SD	*N*	M	SD
Total Scores	1473	1401.5	24.4	960	1392.4	26.5
Assessment and Diagnosis sub-score	1473	61.3	10.8	960	57.5	11.5
Management sub-score	1473	56.1	14.2	960	52.6	14.7
Communication Skills sub-score	1473	69.5	9.5	960	65.7	11.4
Type of IMG	Non-CSA			CSA		
	*N*	M	SD	*N*	M	SD
Total Scores	1286	1398.3	25.5	1147	1397.5	25.8
Assessment and Diagnosis sub-score	1286	60.7	11.2	1147	58.8	11.3
Management sub-score	1286	55.6	14.1	1147	53.7	14.8
Communication Skills sub-score	1286	65.7	10.4	1147	70.5	9.9
Type of PGT	No PGT			PGT		
	*N*	M	SD	*N*	M	SD
Total Scores	1631	1397.0	25.7	802	1399.6	25.5
Assessment and Diagnosis sub-score	1631	58.9	11.2	802	61.5	11.1
Management sub-score	1631	54.1	14.4	802	56.0	14.5
Communication Skills sub-score	1631	69.1	10.4	802	65.8	10.2
Language	English			French		
	*N*	M	SD	*N*	M	SD
Total Scores	2323	1398.0	25.6	110	1396.0	27.8
Assessment and Diagnosis sub-score	2323	59.8	11.2	110	59.5	12.7
Management sub-score	2323	54.8	14.4	110	53.8	16.4
Communication Skills sub-score	2323	68.0	10.5	110	67.3	10.3

### Reliability analyses

September 2020 Cronbach’s alpha ranged from 0.63 to 0.71. October 2021 Cronbach’s alpha ranged from 0.68 to 0.72. DC and DA estimates were also calculated by test form. September 2020 DC values ranged from 0.86 to 0.88 and DA values ranged from 0.90 to 0.92. October 2021 DC values ranged from 0.84 to 0.90 and DA values ranged from 0.89 to 0.93.

### Validity analyses

The first set of analyses were t-tests for the total scores for the NAC Examination for 4 demographic groups defined above (see Table 1 for descriptive statistics). For gender (based on unequal variances), the average score for women was significantly greater than that for men at the *p* < .01 level, t (1925.7) = 8.57, *p* < .0001, 99% CI [6.4, 11.9], and a Cohen’s d of 0.36. For type of CSA (based on equal variances), the average total score was not significantly different by group at the *p* < .01 level, t (2431) = 0.74, *p* = .46, 99% CI [-1.9, 3.5]. For type of PGT (based on equal variances), the difference in average total score was not statistically significant at the *p* < .01 level, t (2431) = -2.34, *p* = .02, 99% CI [-5.4, 0.3]. For language (based on equal variances), the average difference in total scores for those who took the assessment in English or French was not statistically significant at the *p* < .01 level, t (2431) = 0.79, *p* = .43, 99% CI [-4.5, 8.5].

The second set of analyses consisted of four separate MANOVAs (using Wilks’ Lambda) where demographic groupings were the independent variables, and the 3 NAC Examination sub-scores (Assessment and Diagnosis, Management and Communication Skills) were the dependent variables (see Table 2 for descriptive statistics). The MANOVA for gender was statistically significant at the *p* < .01 level, F (3, 2429) 31.42. All 3 step-down analyses for the separate sub-scores were statistically significant at *p* < .01 level, with Communication Skills effect size was the largest (Cohen’s d = 0.36), Assessment and Diagnosis effect size was the second largest (Cohen’s d = 0.34), and Management was the smallest (Cohen’s d = 0.25). For all 3 sub-scores, female candidates had higher average performance than male candidates.

The MANOVA for type of CSA was statistically significant at the *p* < .01 level, F (3, 2429), 118.36. All step-down analyses were statistically significant at the *p* < .01 level, with Communication Skills effect size was the largest (Cohen’s d = 0.47), Assessment and Diagnosis effect size was the second largest (Cohen’s d = 0.17), and Management the smallest (Cohen’s d = 0.13). The average Communication Skills sub-score was higher for those candidates who were CSAs; for Assessment and Diagnosis and Management candidates who were not CSAs had higher average sub-scores.

The MANOVA for type of PGT was statistically significant at the *p* < .01 level, F (3, 2429), 68.38. All step-down analyses were statistically significant at the *p* < .01 level, with Communication Skills effect size was the largest (Cohen’s d = 0.32), Assessment and Diagnosis effect size was the second largest (Cohen’s d = 0.23), and Management was the smallest (Cohen’s d = 0.13). The average Communication Skills sub-score was higher for those candidates without PGT; Assessment and Diagnosis and Management had higher average sub-scores for those candidates with PGT. The MANOVA for language was not statistically significant at the *p* < .01 level, F (3, 2429), 0.30.

The criterion validity evidence is presented in Table 3 as the correlation coefficients for the total scores and sub-scores for both the NAC Examination and MCCQE Part I. All the correlations were statistically significant. The NAC Examination total score and sub-score correlations were large, and the individual sub-score correlations were small to moderate. The correlation between the NAC Examination total score and MCCQE Part I total score was moderate at *r* = .52; the NAC Examination total score and MCCQE Part I sub-score correlations were moderate for Health Promotion and Illness Prevention, Acute Care, Chronic Care, Assessment and Diagnosis, and Management. The correlations were lower for Psychosocial Aspects, Communication and Professional Behaviours. In general, The NAC Examination sub-scores were less highly associated with the MCCQE Part I Psychosocial Aspects, Communication, and Professional Behaviours sub-scores. The highest correlation (*r* = .49) was between the NAC Examination Assessment and Diagnosis sub-score and the MCCQE Part I Assessment and Diagnosis sub-score.

**Table 3:** Correlations for NAC Examination and MCCQE Part I total scores and sub-scores

		NAC Examination				MCCQE Part I								
		1	2	3	4	5	6	7	8	9	10	11	12	13
NAC Examination	1. Total Scores	-												
	2. Assessment and Diagnosis	0.94*	-											
	3. Management	0.79*	0.64*	-										
	4. Communication Skills	0.70*	0.56*	0.48*	-									
MCCQE Part I	5. Total Scores	0.52*	0.48*	0.40*	0.39*	-								
	6. Health Promotion and Illness Prevention	0.41*	0.37*	0.34*	0.31*	0.78*	-							
	7. Acute Care	0.48*	0.44*	0.37*	0.33*	0.91*	0.62*	-						
	8. Chronic Care	0.46*	0.43*	0.37*	0.33*	0.88*	0.58*	0.72*	-					
	9. Psychosocial Aspects	0.29*	0.23*	0.20*	0.31*	0.61*	0.44*	0.43*	0.46*	-				
	10. Assessment and Diagnosis	0.49*	0.46*	0.38*	0.35*	0.93*	0.67*	0.89*	0.85*	0.49*	-			
	11. Management	0.47*	0.44*	0.38*	0.34*	0.90*	0.74*	0.82*	0.80*	0.50*	0.74*	-		
	12. Communication	0.31*	0.26*	0.24*	0.32*	0.60*	0.55*	0.48*	0.47*	0.56*	0.47*	0.45*	-	
	13. Professional Behaviours	0.26*	0.22*	0.21*	0.25*	0.58*	0.53*	0.46*	0.44*	0.61*	0.44*	0.46*	0.36*	-

## Discussion

The use of OSCEs for the certification and licensure of physicians’ dates back 30 years [3]. These performance-based assessments measure what candidates can do, albeit in a simulated environment. The COVID-19 pandemic forced many testing organizations to cancel or modify their performance assessments. For those organizations that modified their assessments, both in terms of administrative protocols and content, it is important to gather additional evidence to support the validity of any inferences based on examination scores. Given that other interruptions to testing could occur in the future, it is also important to document changes in administrative protocols, including those that may have some impact, both positive and negative, on the quality of the assessment. In this study, we gathered evidence to support the psychometric adequacy of the NAC Examination scores for administrations taken under COVID-19 conditions. The results of this study indicated that the NAC Examination had reliable total scores and pass/fail decisions. Moreover, expected differences in total scores and sub-scores for defined groups were consistent with previous literature [19, 35]. The internal relationships amongst NAC Examination sub-scores and their external relationships with the MCCQE Part I supported both discriminant and criterion-related validity arguments. Overall, the changes made to the NAC Examination do not represent threats to the validity of the score interpretations.

With the numerous modifications to the NAC Examination, including the touchless physical examination, other potential sources of measurement error come into play. However, the reliability estimates using Cronbach’s alpha and DC and DA values for pass/fail decisions indicated that the scores are reliable and that consistent pass/fail decisions are being made. Furthermore, the reliability estimates were similar to those found for OSCEs of similar length, and comparable to values observed on NAC Examination test forms administered prior to the COVID-19 pandemic [36]. The DC and DA values were a bit higher than those found before COVID-19 administrations, but they can be influenced by both fewer candidates near the cut score and the overall reliability of the test form. For a decision/interpretation validity framework, the NAC Examination used similar approaches to establish the cut score before and after COVID-19, following best practices [28, 37]. Overall, the NAC Examination modifications yielded scores with acceptable levels of measurement error.

Although the modified NAC Examination scores were reliable, this does not provide concrete evidence that we are measuring the intended abilities. To investigate this, we compared the performances of defined groups. We found that, on average, females outperformed males on Communications skills. As has been documented in other studies, females tend to outperform males on clinical skills assessments, more so for communication [19, 35]. When looking at actual practice data, female physicians have been found to be, on average, better communicators and therefore more likely to obtain more relevant data from patients [38]. We also found that CSAs had better Communication skills than non-CSAs. Since these individuals would have experience in the Canadian education system, one would expect their communication skills, which may be dependent on language proficiency, to be more advanced. It was interesting that non-CSAs, on average, had higher Assessment and Diagnosis and Management sub-scores. This may reflect the fact that non-CSAs have more clinical experience, some having completed part or all of their residency training programs. Evaluating PGT experience, as expected, candidates with prior PGT outperformed those who did not both in Assessment and Diagnosis and Management. Our final comparison looked at performance by language of administration. Given that the English and French NAC Examinations are constructed using the same blueprint to be of comparable difficulty, and there is no reason to believe that the English and French candidates have different abilities, our non-significant finding eliminates one potential threat to the validity of the test scores.

We also quantified the internal associations between the NAC score and sub-scores and their relationships with the scores for an assessment measuring different constructs (MCCQE Part I). The highest criterion-related validity coefficient was between the NAC Examination total score and the Assessment and Diagnosis sub-score for the NAC Examination. From a blueprint perspective, the Assessment and Diagnosis category is the most heavily weighted category, where approximately 70% of the exam content is allocated. The correlations between the NAC Examination sub-scores were moderately high, showing that there was some overlap in the constructs being measured.

The highest correlations for the MCCQE Part I total score were with Assessment and Diagnosis and Acute Care and Management sub-scores of the NAC Examination. The range of correlations between NAC Examination scores and MCCQE Part I scores was expected given the blueprints for both exams share a fair amount of overlap [26, 29]. There are, however, several unique sub-scores on the MCCQE Part I, such as Health Promotion and Illness Prevention, Psychosocial Aspects, and Professional Behaviours. The lower correlations of NAC Examination scores with these dimensions indicate that the two assessments measure different constructs, not unexpected given these constructs are not represented on the NAC Examination blueprint. While we found that NAC Examination Communication score was only moderately associated with the MCCQE Part I Communication sub-score, candidates taking the NAC Examination must demonstrate communication skills as opposed to knowing communication principles (MCCQE Part I). In general, it would be expected that the scores on a written examination would not have very high correlations with a performance-based examination given the different competencies being demonstrated and evaluated.

There is one limitation to interpretation of these correlations, in that candidates challenging the MCCQE Part I may be taking the examination at a different point in time. These correlations may be higher if the two examinations were routinely taken in a short time frame. This may be due to the availability of the MCCQE Part I being available in up to 80 countries and the NAC Examination being offered only within Canada. Candidates who do not pass the MCCQE Part I may also forego taking the NAC Examination as their application for residency may not be competitive. The associations between scores provide some evidence to support the construct and criterion-related validity of the modified NAC Examination.

Some organizations successfully offered virtual performance assessments, generally with smaller candidate numbers for final year medical school examinations [39–44]. Often these assessments were simply an oral examination with small candidate numbers (under 50 candidates) conducted using communication applications such as Zoom or Microsoft Teams, where no physical examination and sometimes no history taking, or communication skills were assessed. Others encountered numerous challenges, potentially compromising the validity of the scores [45]. Even for those organizations that successfully offered a “hands off” assessment, questions concerning the nature of the constructs being measured remain; for example, it is unclear if in-person communication is the same as communication over an electronic platform. Some organizations converted to a virtual platform with larger candidate volumes, but this came with delays and postponing of candidate examination results across several years [46, 47]. This study has outlined that with larger candidate numbers, modifications to existing in-person OSCEs were possible.

## Future considerations

Physical examinations that were “hands-on” were reintroduced to the NAC Examination in May 2023 with masks during the encounters. During the administration of the modified NAC Examination, true assessment of physical examination skills was replaced by an assessment of clinical rationale for specific examination maneuvers. In the end, the assessment of why the candidate wanted to perform specific maneuvers was considered a net gain in that, going forward, the MCC is developing physical examination stations with a hybrid approach, involving a hands-on physical examination and augmenting it with some of the clinical reasoning facets used for the touchless version of the examination. It may be warranted to evaluate long-term outcomes on how the adjustments to the NAC Examination impacted the skills being measured on the adjusted NAC Examination from a program perspective.

Shifting all training, orientation and staff meetings to a virtual platform, with the ability to conduct site meetings more frequently, provided for a greater exchange of information and adoption of best practices. These meetings likely would not have been implemented outside of the urgency of reconfiguring the exam during the pandemic. The COVID-19 pandemic accelerated a positive shift to online educational meetings. The MCC will continue with these virtual sessions at peak preparation times.

## Conclusions

While the modifications to the NAC Examination yielded reliable scores and pass/fail decisions, and some evidence to support their validity, the assessment was different, both in terms of administration and content. With respect to administration, the practice of medicine at the time was being carried out under PPE conditions, thus enhancing the fidelity of the assessment. Given that the reliability of the scores was similar to those for pre-COVID administrations, it is reasonable to presume that the online training of SPs and physician examiners was adequate. Finally, the “touchless” physical examination, a necessary modification at the time, cannot be used to measure a candidate’s ability to evaluate objective anatomic findings through the use of palpation, percussion, and auscultation. It can, however, be employed to measure clinical reasoning related to physical examination skills, a necessary competency for the practice of medicine. All in all, the COVID-19 pandemic provided the impetus to make changes to OSCEs, many of which were positive. Going forward, the MCC and other organizations now have the expertise and knowledge base to appropriately modify their assessments should another health crisis or other type of examination interruption occur.

Gathering evidence to support the validity of examination scores, or any inferences we make based on the examination scores, is never complete [48]. While we do provide some evidence to support the reliability and validity of the NAC Examination as administered under COVID-19 conditions, additional investigations are warranted. In moving to a “touchless” physical examination, a different construct is being measured. However, one would still expect that knowledge of which physical examination maneuvers were appropriate should be related to the ability to perform other physical examination skills. This could be studied in the future. Given the artificial nature of the simulation environment, it is important to know whether NAC Examination performance relates to performance with ‘real’ patients. While the relationships between OSCE performance and patient care have been studied elsewhere [19], this has not been done specifically for the NAC Examination. The MCC, like most testing organizations, is dedicated to providing data to support the valid use of its examinations.

## Data Availability

The datasets generated and/or analyzed during the current study are not publicly available due to the confidentiality of the examination results of the candidates. De-identified data can be provided by reasonable request to Andrea Gotzmann at agotzmann@mcc.ca.

## Footnotes

1 Data between September 2020 through 2021 was used for this study, as these data were approved in our ethics application.

2 Data between September 2020 through 2021 was used for this study, as these data were approved in our ethics application.

## Abbreviations

NAC: National Assessment Collaboration
OSCE: Objective Structured Clinical Examination
IMG: International Medical Graduate
CSA: Canadians Studying Abroad
PGT: Postgraduate training
MCCQE: Medical Council of Canada Qualifying Examination
SP: Standardized participants
MCC: Medical Council of Canada
USMLE: United States Medical Licensing Examination
CS: Clinical Skills (CS) in the context of the USMLE Step 2 Clinical Skills examination
NBOME: National Board of Osteopathic Medical Examiners
PE: Performance Evaluation (PE) in the context of the COMLEX-USA-Level 2
PPE: Personal protective equipment
SME: Subject matter experts
DC: Decision consistency
DA: Decision accuracy
MANOVA: Multivariate analyses of variance
SD: Standard deviation
COVID-19: Coronavirus disease of 2019
ANOVA: Analysis of variance

## References

[1] Harden RMG, Stevenson M, Downie WW, Wilson GM. Assessment of clinical competence using objective structured examination. Br Med J. 1975;1(5955):447–51. 10.1136/bmj.1.5955.447 1115966 10.1136/bmj.1.5955.447 PMC1672423

[2] Khan KZ, Ramachandran S, Gaunt K, Pushkar P. The objective structured clinical examination (OSCE): AMEE Guide 81. Part I: an historical and theoretical perspective. Med Teach. 2013;35(9). 10.3109/0142159X.2013.818634 10.3109/0142159X.2013.818634 23968323

[3] Boulet JR, Smee SM, Dillon GF, Gimpel JR. The use of standardized patient assessments for certification and licensure decisions. Simul Healthc. 2009;4(1):35–42. 10.1097/SIH.0b013e318182fc6c 19212249 10.1097/SIH.0b013e318182fc6c

[4] Brailovsky Ca, Grand’Maison P, Lescop J. A large-scale multicenter objective structured clinical examination for licensure. Acad Med. 1992;67(10):S37–9. 10.1097/00001888-199210000-00032 1388549 10.1097/00001888-199210000-00032

[5] Lee YS. OSCE for the medical licensing examination in Korea. Kaohsiung J Med Sci. 2008;24(12):646–50. 10.1016/S1607-551X(09)70030-0 19251560 10.1016/S1607-551X(09)70030-0 PMC11917856

[6] Berendonk C, Schirlo C, Balestra G, et al. The new final clinical skills examination in human medicine in Switzerland: essential steps of exam development, implementation and evaluation, and central insights from the perspective of the national working group. GMS Z Med Ausbild. 2015;32(4):1–13. 10.3205/zma000982 10.3205/zma000982 PMC4606485 26483853

[7] Hodges BD, Hollenberg E, McNaughton N, Hanson MD, Regehr G. The psychiatry OSCE: a 20-year retrospective. Acad Psychiatry. 2014;38(1):26–34. 10.1007/s40596-013-0012-8 24449223 10.1007/s40596-013-0012-8

[8] Tavares W, Brydges R, Myre P, et al. Applying Kane’s validity framework to a simulation based assessment of clinical competence. Adv Heal Sci Educ. 2018;23(2):1–16. 10.1007/s10459-017-9800-3 10.1007/s10459-017-9800-3 29079933

[9] Hodges B. Validity and the OSCE. Med Teach. 2003;25(3):250–4. 10.1080/01421590310001002836 12881045 10.1080/01421590310001002836

[10] Singer PA, Cohen R, Robb A, Rothman A. The ethics objective structured clinical examination. J Gen Intern Med. 1993;8(1):23–8. 10.1007/BF02600289 8419558 10.1007/BF02600289

[11] Cunnington JPW, Neville AJ, Norman GR. The risk of thoroughness: reliability and validity of global rating and checklists in an OSCE. Adv Heal Sci Educ. 1997;1(3):227–33. 10.1007/BF00162920 10.1007/BF00162920 24179023

[12] Turner JL, Dankoski ME. Objective structured clinical exams: a critical review. Fam Med. 2008;40(8):574–8. http://www.ncbi.nlm.nih.gov/pubmed/18988044. Accessed September 4, 2019. 18988044

[13] Quero Munoz L, O’Byrne C, Pugsley J, Austin Z. Reliability, validity, and generalizability of an objective structured clinical examination (OSCE) for assessment of entry-to-practice in pharmacy. Pharm Educ. 2005;5(1):33–43. 10.1080/15602210400025347 10.1080/15602210400025347

[14] Dong T, Swygert KA, Durning SJ, et al. Validity evidence for medical school OSCEs: associations with USMLE^®^ Step assessments. Teach Learn Med. 2014;26(4):379–86. 10.1080/10401334.2014.960294 25318034 10.1080/10401334.2014.960294

[15] Felthun JZ, Taylor S, Shulruf B, Allen DW. Assessment methods and the validity and reliability of measurement tools in online objective structured clinical examinations: a systematic scoping review. J Educ Eval Health Prof. 2021;18. 10.3352/JEEHP.2021.18.11 10.3352/jeehp.2021.18.11 PMC8212027 34058802

[16] Pugh DM, Wood TJ, Boulet JR. Assessing procedural competence: validity considerations. Simul Healthc. 2015;10(5):288–94. 10.1097/SIH.0000000000000101 26426560 10.1097/SIH.0000000000000101

[17] Hastie MJ, Spellman JL, Pagano PP, Hastie J, Egan BJ. Designing and implementing the objective structured clinical examination in anesthesiology. Anesthesiology. 2014;120(1):196–203. 10.1097/ALN.0000000000000068 24212197 10.1097/ALN.0000000000000068

[18] Wenghofer E, Boulet J. Medical Council of Canada Qualifying Examinations and performance in future practice. Can Med Educ J. 2022;13(4):53–61. 10.36834/cmej.73770 36091726 10.36834/cmej.73770 PMC9441123

[19] Boulet JR, McKinley DW. Investigating gender-related construct-irrelevant components of scores on the written assessment exercise of a high-stakes certification assessment. Adv Heal Sci Educ. 2005;10(1):53–63. 10.1007/s10459-004-4297-y 10.1007/s10459-004-4297-y 15912284

[20] Craig B, Wang X, Sandella J, Tsai T-HH, Kuo D, Finch C. Examining concurrent validity between COMLEX-USA Level 2-Cognitive evaluation and COMLEX-USA Level 2-Performance evaluation. J Osteopath Med. 2021;121(8):4–8. 10.1515/jom-2021-0007 10.1515/jom-2021-0007 33979903

[21] McKinley DW, Boulet JR, Hambleton RK. A work-centered approach for setting passing scores on performance-based assessments. Eval Heal Prof. 2005;28(3):349–69. 10.1177/0163278705278282 10.1177/0163278705278282 16123262

[22] Bobos P, Pouliopoulou DVS, Harriss A, Sadi J, Rushton A, MacDermid JC. A systematic review and meta-analysis of measurement properties of objective structured clinical examinations used in physical therapy licensure and a structured review of licensure practices in countries with well-developed regulation systems. PLoS ONE. 2021;16(8 August). 10.1371/journal.pone.0255696 10.1371/journal.pone.0255696 PMC8330929 34343213

[23] United States Medical Licensing Examination. Work to relaunch USMLE Step 2 CS discontinued | USMLE. Announcements. Published 2021. Accessed December 7. 2022. https://www.usmle.org/work-relaunch-usmle-step-2-cs-discontinued

[24] National Board of Osteopathic Medical Examiners. NBOME Board Accepts Final Report from Special Commission — NBOME. Accessed December 18. 2022. https://www.nbome.org/news/final-report-from-special-commission/

[25] Medical Council of Canada. The MCC ceases delivery of the MCCQE Part II. Published 2021. Accessed November 4. 2021. https://mcc.ca/news/mcc-ceases-delivery-of-the-mccqe-part-ii/?cn-reloaded=1

[26] Medical Council of Canada. NAC Overview | Medical Council of Canada. Accessed April 20. 2022. https://mcc.ca/examinations/nac-overview/

[27] Kolen MJ, Brennan RL. Test equating, scaling, Link methods Pract Third Ed. Springer; 2014. 10.1007/978-1-4939-0317-7/COVER

[28] Medical Council of Canada. Technical Report on the 2020 Standard-Setting Exercise for the NAC Examination. 2020. Accessed July 12, 2020. https://mcc.ca/research-and-development/technical-reports/

[29] Medical Council of Canada. MCCQE Part I | Medical Council of Canada. Accessed December 18. 2022. https://mcc.ca/examinations/mccqe-part-i/

[30] Medical Council of Canada. Blueprint | News Tags | Medical Council of Canada. Accessed December 18. 2022. https://www.mcc.ca/tags/blueprint/

[31] Haertel EH. Reliability. In: Brennan RL, ed. Educational Measurement. 4th ed. Praeger; 2006:65–110.

[32] Livingston SA, Lewis C. Estimating the consistency and accuracy of classifications based on test scores. J Educ Meas. 1995;32(2):179–97. 10.1111/j.1745-3984.1995.tb00462.x 10.1111/j.1745-3984.1995.tb00462.x

[33] Tabachnick BG, Fidell LS. Using Multivariate statistics. 7th ed. Pearson; 2019. https://lccn.loc.gov/2017040173

[34] Cohen J. Statistical Power Analysis for the behavioral sciences. 2nd ed. Lawrence Erlbaum Associates; 1988. 10.4324/9780203771587

[35] Weidner AC, Gimpel JR, Boulet OR, Solomon M. Using standardized patients to assess the communication skills of graduating physicians for the comprehensive osteopathic medical licensing examination (COMLEX) level 2-Performance evaluation (level 2-PE). Teach Learn Med. 2010;22(1):8–15. 10.1080/10401330903445604 20391277 10.1080/10401330903445604

[36] Medical Council of Canada. NAC Examination annual technical report - September 2020. 2020. Accessed July 12, 2020. https://mcc.ca/research-and-development/technical-reports/

[37] Cizek GJ, Bunch MB. Standard setting: a guide to establishing and evaluating performance standards on tests. Published Online 2007:352.

[38] Tanne JH. Women doctors are better communicators. BMJ Br Med J. 2002;325(7361):408. Accessed December 18, 2022. https://www.ncbi.nlm.nih.gov/pmc/articles/PMC1123938/#:~:text=Female doctors spent an average,more actively sought patient input

[39] Quinlin L, Clark Graham M, Nikolai C, Teall AM. Development and implementation of an e-visit objective structured clinical examination to evaluate student ability to provide care by telehealth. J Am Assoc Nurse Pract. 2020;00(00):1. 10.1097/jxx.0000000000000409 10.1097/jxx.0000000000000409 32304481

[40] Arrogante O, López-Torre EM, Carrión-García L, Polo A, Jiménez-Rodríguez D. High-fidelity virtual objective structured clinical examinations with standardized patients in nursing students: an innovative proposal during the covid-19 pandemic. Healthc. 2021;9(3). 10.3390/healthcare9030355 10.3390/healthcare9030355 PMC8004020 33804700

[41] Craig C, Kasana N, Modi A, Virtual. OSCE delivery: the way of the future? Med Educ. 2020;54(12):1185–6. 10.11111/medu.14286 32627218 10.11111/medu.14286

[42] Hopwood J, Myers G, Sturrock A. Twelve tips for conducting a virtual OSCE. Med Teach. 2021;43(6):633–6. 10.1080/0142159X.2020.1830961 33078984 10.1080/0142159X.2020.1830961

[43] Boyle JG, Colquhoun I, Noonan Z, McDowall S, Walters MR, Leach JP. Viva La VOSCE? BMC Med Educ. 2020;20:514. 10.1186/s12909-020-02444-3 33334327 10.1186/s12909-020-02444-3 PMC7746425

[44] Blythe J, Patel NSA, Spiring W, Easton G, Evans D, Meskevicius-Sadler E, Noshib H, Gordon H. Undertaking a high stakes virtual OSCE (VOSCE) during Covid-19. BMC Med Educ. 2021;21:221. 10.1186/s12909-021-02660-5 33879139 10.1186/s12909-021-02660-5 PMC8057662

[45] Zulkifly HH, Zaki IAH, Karuppannan M, Noordin ZM, Virtual OSCE. Experience and challenges with a large cohort of pharmacy students. Pharm Educ. 2022;22(1):23–32. 10.46542/pe.2022.221.2332 10.46542/pe.2022.221.2332

[46] Lemire F, Fowler N, Kvern B. CFPC examinations and COVID-19. Can Fam Physician. 2020;66(8) Accessed June 5, 2024. https://www.ncbi.nlm.nih.gov/pmc/articles/PMC7430795/PMC7430795 32817043

[47] Kim E, Update, #13. COVID-19 and Exams. Accessed June 5, 2024. https://residentdoctors.ca/news-events/news/update-13-covid-19-and-exams/

[48] Cook DA, Hatala R. Validation of educational assessments: a primer for simulation and beyond. Adv Simul. 2016;1(1):1–12. 10.1186/s41077-016-0033-y 10.1186/s41077-016-0033-y PMC5806296 29450000

